# Supplementary material for: Detection and Characterization of Methylated Circulating Tumor DNA in Gastric Cancer
Source: Int J Mol Sci. 2024 Jul 5;25(13):7377. doi: 10.3390/ijms25137377 (PMC11242052; doi:10.3390/ijms25137377)
Supplement: Supplementary file 1 [file ijms-25-07377-s001.zip › ijms-3005315-supplementary.pdf]

<Supplementary data>

Supplementary Table S1. Number of DMR and association with promoter.

# Case	# Ctrl.	# All DMRs	# Hyper methylations	# Hypo methylations	# Hyper methylations On Promoter	# Hypo methylations On Promoter
22 (GC Plasma)	40 (Healthy Plasma)	316,507	257,323	59,184	8,476	1,235
22 (GC Tissue)	40 (Healthy Plasma)	1,224,857	956,613	268,244	22,965	8,957

Promoter region is defined as ±1kb region from transcription start site.

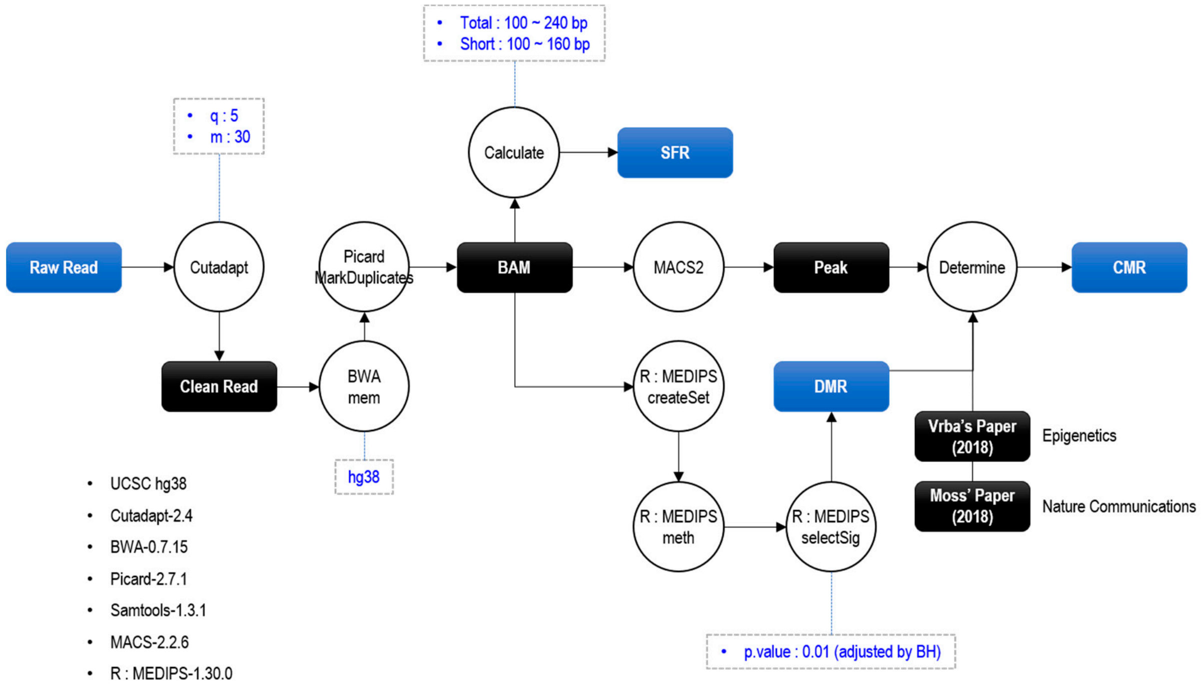

Supplementary Figure S1. Flowchart for DMR, CMR and SFR sorting.

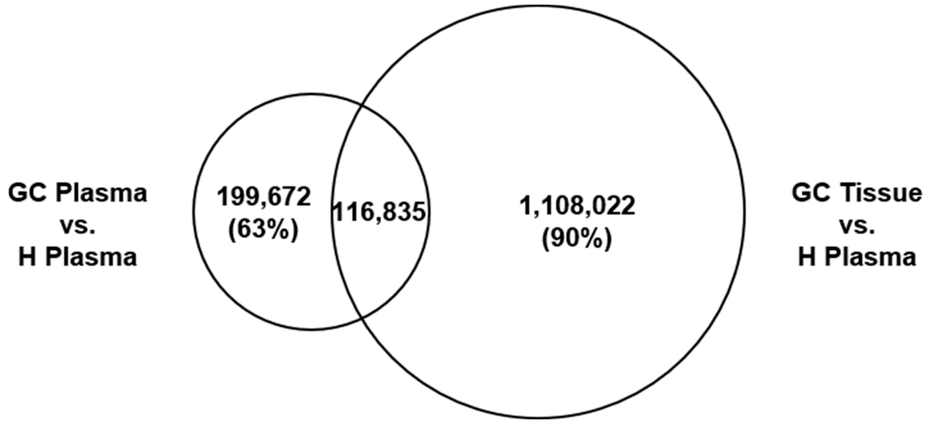

Supplementary Figure S2. Venn diagram shows count of DMR and CMR between patient tissue and plasma. For DMR, plasmas and tissues from patients were compared with the healthy group. Then, CMR was obtained by comparing plasma and tissue of GC patients.

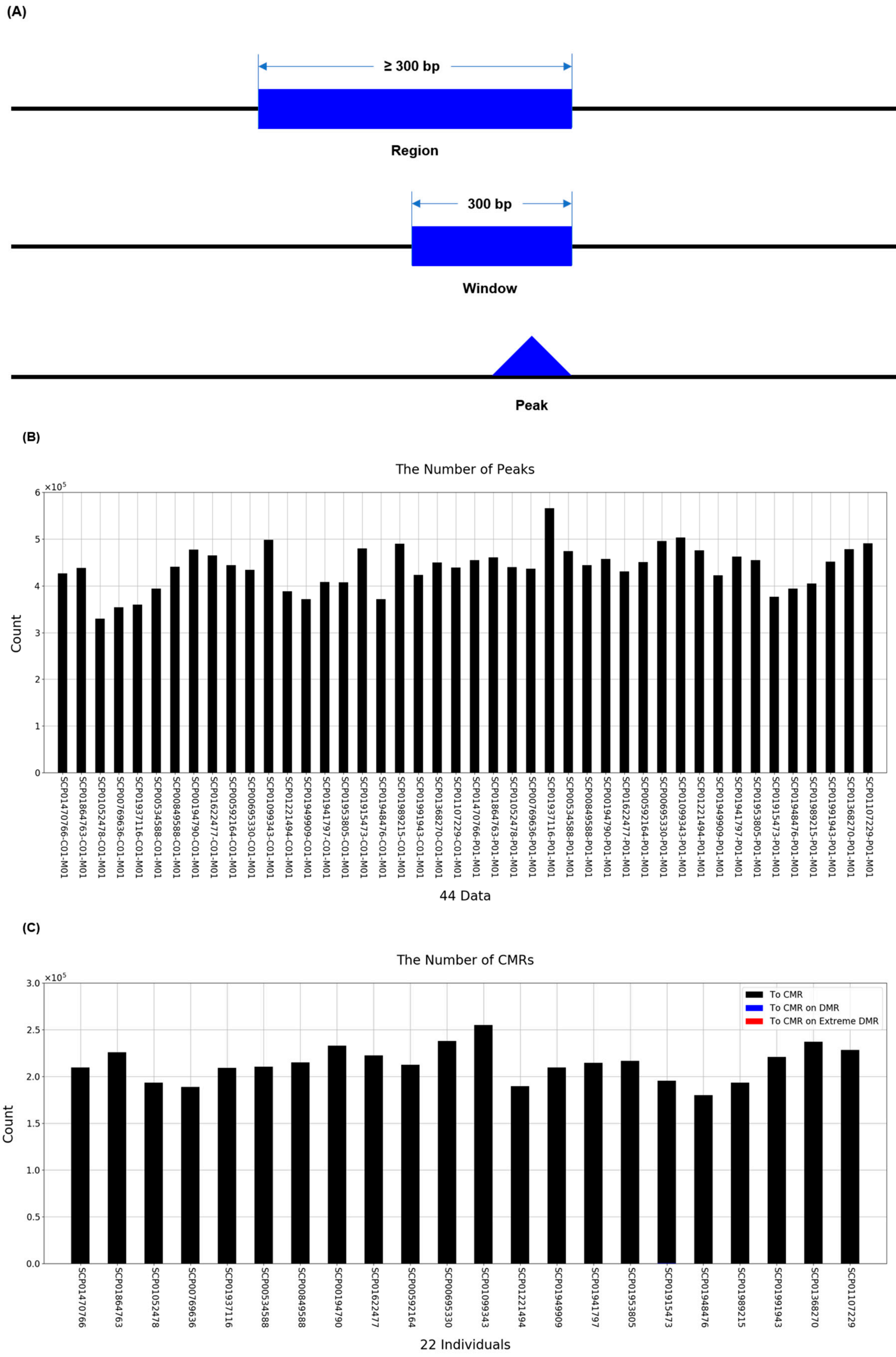
(A)
≥ 300 bp
Region
300 bp
Window
Peak
(B)
The Number of Peaks
×10^5
Count
0
1
2
3
4
5
6
SCP01470766-C01-M01
SCP01864763-C01-M01
SCP01052478-C01-M01
SCP00769636-C01-M01
SCP01937116-C01-M01
SCP00534588-C01-M01
SCP00849588-C01-M01
SCP00194790-C01-M01
SCP01622477-C01-M01
SCP00592164-C01-M01
SCP00695330-C01-M01
SCP01099343-C01-M01
SCP01221494-C01-M01
SCP01949909-C01-M01
SCP01941797-C01-M01
SCP01953805-C01-M01
SCP01915473-C01-M01
SCP01948476-C01-M01
SCP01989215-C01-M01
SCP01991943-C01-M01
SCP01368270-C01-M01
SCP01107229-C01-M01
SCP01470766-P01-M01
SCP01864763-P01-M01
SCP01052478-P01-M01
SCP00769636-P01-M01
SCP01937116-P01-M01
SCP00534588-P01-M01
SCP00849588-P01-M01
SCP00194790-P01-M01
SCP01622477-P01-M01
SCP00592164-P01-M01
SCP00695330-P01-M01
SCP01099343-P01-M01
SCP01221494-P01-M01
SCP01949909-P01-M01
SCP01941797-P01-M01
SCP01953805-P01-M01
SCP01915473-P01-M01
SCP01948476-P01-M01
SCP01989215-P01-M01
SCP01991943-P01-M01
SCP01368270-P01-M01
SCP01107229-P01-M01
44 Data
(C)
The Number of CMRs
×10^5
To CMR
To CMR on DMR
To CMR on Extreme DMR
Count
0.0
0.5
1.0
1.5
2.0
2.5
3.0
SCP01470766
SCP01864763
SCP01052478
SCP00769636
SCP01937116
SCP00534588
SCP00849588
SCP00194790
SCP01622477
SCP00592164
SCP00695330
SCP01099343
SCP01221494
SCP01949909
SCP01941797
SCP01953805
SCP01915473
SCP01948476
SCP01989215
SCP01991943
SCP01368270
SCP01107229
22 Individuals

(D)

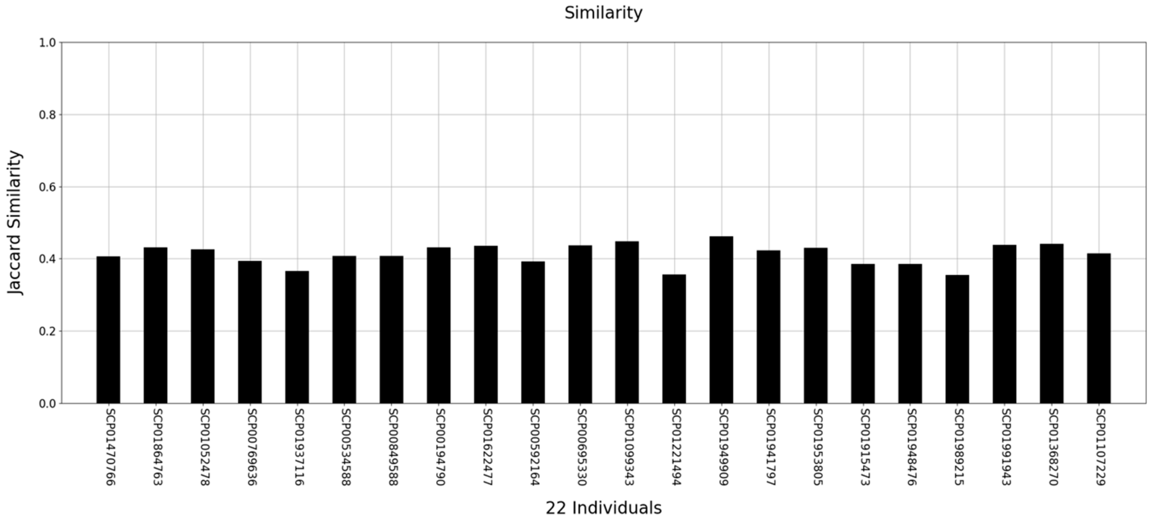

Supplementary Figure S3. Determination and calculation of CMR. (A) Schematic diagram and definitions of regions, windows, and peaks of CMR. (B) Graph shows the peak number of 22 pairs of cancer patient samples individually. (C) and (D) Graphs show number and similarity of CMRs between tissue and plasma from each patient. The numbers of the blue and red bars are so small that they are barely visible on the graph.
